# The Missing Heritability of Sporadic Frontotemporal Dementia: New Insights from Rare Variants in Neurodegenerative Candidate Genes

**DOI:** 10.3390/ijms20163903

**Published:** 2019-08-10

**Authors:** Miriam Ciani, Cristian Bonvicini, Catia Scassellati, Matteo Carrara, Carlo Maj, Silvia Fostinelli, Giuliano Binetti, Roberta Ghidoni, Luisa Benussi

**Affiliations:** 1 Molecular Markers Laboratory, IRCCS Istituto Centro San Giovanni di Dio Fatebenefratelli, 25125 Brescia, Italy; 2 Service of Statistics, IRCCS Istituto Centro San Giovanni di Dio Fatebenefratelli, 25125 Brescia, Italy; 3 Institute of Genomic Statistics and Bioinformatics, University of Bonn, 53127 Bonn, Germany

**Keywords:** frontotemporal dementia, sporadic cases, early onset, next generation sequencing, genetic rare variants, neurodegenerative disease

## Abstract

Frontotemporal dementia (FTD) is a common form of dementia among early-onset cases. Several genetic factors for FTD have been revealed, but a large proportion of FTD cases still have an unidentified genetic origin. Recent studies highlighted common pathobiological mechanisms among neurodegenerative diseases. In the present study, we investigated a panel of candidate genes, previously described to be associated with FTD and/or other neurodegenerative diseases by targeted next generation sequencing (NGS). We focused our study on sporadic FTD (sFTD), devoid of disease-causing mutations in *GRN*, *MAPT* and *C9orf72*. Since genetic factors have a substantially higher pathogenetic contribution in early onset patients than in late onset dementia, we selected patients with early onset (<65 years). Our study revealed that, in 50% of patients, rare missense potentially pathogenetic variants in genes previously associated with Alzheimer’s disease, Parkinson disease, amyotrophic lateral sclerosis and Lewy body dementia (*GBA*, *ABCA7*, *PARK7*, *FUS*, *SORL1*, *LRRK2*, *ALS2*), confirming genetic pleiotropy in neurodegeneration. In parallel, a synergic genetic effect on FTD is suggested by the presence of variants in five different genes in one single patient. Further studies employing genome-wide approaches might highlight pathogenic variants in novel genes that explain the still missing heritability of FTD.

## 1. Introduction

Frontotemporal dementia (FTD) is an adult-onset neurodegenerative disorder affecting the frontal and temporal lobes of the brain that is characterized by a variety of symptoms, including behavioral disturbances and alterations in different language properties [1,2,3,4,5]. Specifically, behavioral abnormalities are prominent in the behavioral variant (bvFTD) [4], while language deficits are observed in the primary progressive aphasias (PPAs), further divided in the progressive non-fluent agrammatic variant and the semantic variant [3]. In addition, movement disorders can be observed in the clinical phenotype [6,7].

Genetic aetiology has been revealed in 30–40% of FTD patients that show a positive family history of dementia [8]. In many of these cases, FTD is inherited with an autosomal-dominant pattern, and disease-causing mutations have been recognized in several genes (http://www.molgen.ua.ac.be/ADMutations/; https://www.alzforum.org/), among which the microtubule associated protein Tau (*MAPT*), the progranulin (*GRN*), and the chromosome 9 open reading frame 72 (*C9orf72*) genes represent the main key-players of inherited FTD [9,10]. In addition, the valosin (*VCP*), the charged multivesicular body protein 2B (*CHMP2B*), the TAR DNA binding protein (*TARDP*), the triggering receptor expressed on myeloid cells 2 (*TREM2*), the ubiquilin 2 (*UBQLN2*), the sequestosome 1 (*SQSTM1*) and the fused in sarcoma (*FUS*) genes are also known for their implication in this disease.

It should be noted that it is increasingly clear that FTD clinical and molecular features are shared with other neurodegenerative disorders, suggesting that different types of dementia may be caused by overlapping genetic factors [11,12,13]. For instance, the *MAPT* pathway is involved in the aetiopathogenesis of Alzheimer’s disease (AD), Parkinson’s disease (PD), parkinsonism, and amyotrophic lateral sclerosis (ALS) [14,15,16]; *GRN* in AD, PD, parkinsonism, motor neuron disease (MND) [17,18,19,20]; and *C9orf72* in ALS, AD, and parkinsonism [21]. This scenario exemplifies the emerging observation of phenotypic pleiotropy, where mutations in the same gene give rise to diverse phenotypes, further increasing the complexity of genotype–phenotype correlation.

Regardless, disease-causing variants detected in these genes are not sufficient to represent the whole genetic background of this disorder [22]. Indeed, there are many sporadic FTD cases (sFTD), in which the disease seems to occur sporadically with an unclear pattern of inheritance [23]. We recently used specific criteria to classify a wide cohort of FTD Italian families into genetic risk categories [9,10]. According to these criteria, 39% of subjects from our cohort were classified as sFTD, and a pathogenic mutation in one of the three common FTD genes (*MAPT*, *GRN*, *C9orf72*) was detected only in the 1.3% of this group, highlighting a “missing heritability” to be explored.

To this aim, we herein performed next generation sequencing (NGS) combined with bioinformatics approaches on a selected cohort of sFTD subjects belonging to our previous characterized FTD cohort [10] in order to detect rare variants, predicted to have a functional effect, in a panel of 43 genes previously associated to neurodegenerative diseases (i.e., FTD, AD, ALS, MND, and PD).

## 2. Results

### 2.1. Genetic Screening and Validation of Rare Genetic Alterations in Sporadic FTD

A cohort of eight sFTD patients was included in this study (Table 1).

To enrich genetic background, early onset patients were selected (onset < 65 years). Starting from the hypothesis that sFTD might be characterized by an impairment of molecular pathways altered in other neurodegenerative disorders, candidate genes implicated not only in FTD but also in other neurodegenerative diseases were selected (Table S1). To facilitate variants filtering and prioritization, we selected coding variants (i.e., ins/del frameshift, stop gain/stop loss and missense variants) and, regarding missense variants, only those with an allele frequency ≤0.01 were considered.

Overall, for all selected genes, no insertion/deletion frameshift or stop gain/stop loss mutations were detected. Moreover, no subject showed rare variants in the known FTD-causing genes (i.e., *CHMP2B*, *TARDBP*, *VCP*, along with *MAPT*, *GRN* and *C9orf72*), and 50% of sFTD patients did not carry any variants in the screened genes (Patients 2, 3, 4, 8). Interestingly, in the remaining 50% (Patients 1, 5, 6, 7), we identified missense variants in seven genes (Table 2).

Specifically, Patient 1 showed a rare missense variant in the glucosylceramidase beta (*GBA*) gene; Patient 5 presented a rare variant in the ATP binding cassette subfamily A member 7 (*ABCA7*) gene; Patient 6 showed a rare variant in the Parkinsonism-associated deglycase 7 (*PARK7*) gene; Patient 7 presented multiple rare variants in five genes, i.e., *ABCA7*, the sortilin related receptor 1 (*SORL1*) gene, the *FUS* gene, the alsin Rho guanine nucleotide exchange factor (*ALS2*) gene, and the leucine rich repeat kinase 2 (*LRRK2*) gene. All the eight variants were confirmed by Sanger sequencing (Figure S1).

### 2.2. In Silico Analyses of Functional Impact of the Identified Rare Variants

We reported the Residual Variation Intolerance Score (RVIS) value of each candidate gene in which a variant was identified (Table 3). In particular, the *ABCA7*, *FUS*, *SORL1*, *LRRK2*, and *ALS2* genes showed negative values of RVIS, indicating their intolerance to functional variation and, thus, a potentially high deleterious impact of the identified mutations; on the contrary, *GBA* and *PARK7* genes were characterized by RVIS > 0, thus representing tolerant genes, with a higher number of common functional variations.

In addition, to evaluate the impact of validated variants on protein conformation and function (deleterious effect), the evolutionary conservation of nucleotide, and amino acid variations, specific bioinformatics programs were used (Table 3). Overall, the pathogenicity of each variant was evaluated considering either the Combined Annotation Dependent Depletion (CADD) and the radial support vector machine (radial SVM), two ensemble scores based on the simultaneous evaluation and integration of different independent bio-informatic scoring tools. In particular, a genetic variant was considered potentially pathogenic if at least one of the two scores was damaging. Based on this in silico assessment, the identified genetic variations in *GBA* and *ABCA7* were scored as potentially damaging according to the radial SVM, whereas *ALS2* was classified as likely pathogenic by the CADD. The other mutations in *FUS*, *LRRK2*, and *PARK7* seemed to be tolerated.

Interestingly, both the ensemble scores classified the *SORL1* variant as putatively pathogenic. Thus, to evaluate the localization and the effect of this variant on the protein, further bioinformatics analyses with Elaspic and PyMol2 were performed. In particular, we found that the c.C2185T (p.R729W) variant fell in a functional domain (VPS10P) and influenced the binding forces and, consequently, the 3D crystal protein structure (Videos S1, S2, S3). Of note, we also found that the *GBA*, *ABCA7* and *PARK7* variants fell into the core of a domain (*GBA*: Glycosyl hydrolase family 30 TIM-barrel domain; *ABCA7*: ABC transporter; *PARK7*: DJ-1/PfpI ) with possible effects on the domain stability, while the *ALS2* variant geell on the surface of a domain (RCC1) responsible for the interface with three ras family small GTPases (RAB5A, RAC1 and RAC1 Isoform 2).

## 3. Discussion

High-throughput sequencing technologies are particularly useful for the study of complex diseases, mainly opening the door to chase for new genetic players and rare coding variants not considered before [24]. Interestingly, several genetic factors for FTD have been revealed, but a large proportion of FTD cases still has an unidentified genetic origin [10,25]. Recent studies have highlighted common pathobiological mechanisms among neurodegenerative diseases, e.g., AD, FTD, Lewy body disease (LBD), and PD [26,27,28,29,30,31,32,33,34]. Thus, in the present study, we selected a panel of candidate genes, previously described to be associated with FTD and/or other neurodegenerative diseases, to be investigated by NGS. We focused our study on sFTD, negative for the presence of disease-causing mutations in *GRN*, *MAPT* and *C9orf72*. Since genetic factors have a substantially higher pathogenetic contribution in early onset patients than in late onset dementia, we selected patients with disease onset before 65 years of age. Overall, our study identified new genetic variants potentially involved in FTD aethiopathogenesis, and it evidenced both a potential pleiotropic and a polygenic effect of genes. Specifically, these potentially pathogenetic variants were located in eight genes: *GBA*, *ABCA7*, *PARK7*, *FUS*, *SORL1*, *LRRK2*, and *ALS2*. With the exclusion of *FUS*, all genes were not previously described to be associated with FTD. However, all these genes play a key role in cellular pathways known to be impaired in FTD.

The *GBA* gene encodes the beta-glucocerebrosidase, an enzyme active in lysosomes which physiologically breaks down the glucocerebroside, a complex component of cellular membrane, into glucose and ceramide [35]. Mutations in *GBA* gene are usually found in patients affected from Gaucher’s disease [36], but they were also identified in PD and LBD patients [37,38,39]. In Gaucher’s disease, a glycolipid storage disorder, pathogenic *GBA* mutations reduce or eliminate the activity of the glucocerebrosidase enzyme, causing an abnormal accumulation of glucocerebroside into lysosomes and, thus, damaging different tissue and organs [35]. In out cohort, Patient 2 showed a known rare non-synonymous variant (rs2230288, p.E278K) in this gene.

Though the RVIS of *GBA* is > 0, the variant herein identified could have a damaging effect as provided by the radial SVM; this variant falls into the core of the glycosyl hydrolase family 30 TIM-barrel domain. Moreover, the *GBA* p.E278K mutation has been already described in literature and suggested as susceptibility variant in PD patients [40]. Specifically, heterozygous *GBA* mutated-PD patients showed an increased disease risk, earlier age at onset, and faster progression. In addition to cognitive decline, alterations in executive functions and language processing were observed in patients carrying this genetic alteration [40,41]. Alterations in the lysosomal pathways have been widely described in sporadic and genetic FTD [42,43,44,45]. Similarly, it is interesting to note that null mutations in *GRN* can cause a lysosomal storage disorder and alterations in lysosomal homeostasis [46,47,48]. All these evidences support a potential pathogenic role of *GBA* in sFTD.

The protein encoded by *ABCA7* is a sphingolipids and cholesterol transporter which has also a role in endocytosis regulation and Aβ clearance [49,50,51]. Previous genetic analyses have identified numerous rare loss of function and missense variants strongly correlated to AD risk [52,53]. Moreover, an enrichment in loss of function *ABCA7* variants was observed in early onset AD patients, supporting its specific involvement in early onset dementia [52,54,55]. Interestingly, the ABCA7 protein binds apolipoprotein (a) and promotes apolipoprotein-mediated phospholipid efflux from cells [49]. An association between the apolipoprotein (a) isoforms and FTD has been reported, indicating a link between this pathway and FTD [56]. Our screening revealed two variants which are unknown in the ExAC database in two different patients (Patient 5: c.C5585A, p.P1862H; Patient 7: c.A5389C, p.N1797H). According to its RVIS value, *ABCA7* is highly intolerant to mutations. In addition, as supported by an in silico analysis (the radial SVM), both identified variants are predicted to be deleterious and located into the core of the ABC transporter, suggesting that these mutations could impact on specific pathways linked to *ABCA7* and could thus cause FTD.

*PARK7* mutations have been described in early-onset forms of PD, both familial and sporadic, as well as in other neurodegenerative disorders where oxidative stress is involved [57]. The *PARK7* gene encodes for a chaperone molecule localized in the nucleus and cytoplasm of both neuronal and glial cells, and it seems to be also implicated in oxidative stress. Thus, experimental evidences suggest that *PARK7* mutations compromise the chaperone function, leading to a toxic buildup of misfolded or damaged proteins and eventually to cell death [58]; other reports described defects in specific oxidative stress processes and alterations in mitochondrial functions [58,59]. Of note, the complex relationship between energy metabolism and neurodegenerative disorders, including FTD, is one of the most studied topics [60,61]. The identified *PARK7* variant (Patient 6: rs45577037, p.T110A) is extremely rare, but it seems to be tolerated, as calculated with the two ensemble scores and as confirmed by the positive RVIS value of the gene. Considering the single bio-informatic scores, it is possible to observe that the nucleotide substitution is on a very conserved position in the evolution and is located into the core of the DJ-1/PfpI domain. Thus, a functional validation of this variant would be necessary to better investigate its pathogenicity.

*FUS* mutations were identified in familial and sporadic ALS and FTD, mainly characterized by an early-onset [28,62,63,64]. Moreover, Bradfield et al. reported a 61-year-old sFTD patient with *FUS* alterations who exhibited both behavioral and language disorders with a rapidly progressive clinical course [65]. The protein encoded by *FUS* is a DNA/RNA binding protein that plays a role in a multitude of critical cellular functions, including gene expression, RNA processing/transport, genomic integrity, and autophagic pathways [66,67]. The majority of disease-causing *FUS* mutations are located in the C-terminus region and seems to disrupt nuclear import, while others inhibit the nuclear import of the protein, leading to cytoplasmic protein inclusions in neurons and glial cells [28,68,69,70]. In this study, Patient 7 showed an extremely rare missense variant (c.G235A, p.G235A) in this gene, resulting tolerated by using bio-informatic tools. However, *FUS* has a negative RVIS value, indicating that genetic alterations might be potentially damaging; thus, a further analysis is needed. 

The *SORL1* gene has been widely described as implicated in late and early onset forms of AD: Studies on large cohorts associated both common and rare variants in this gene with AD, including sporadic forms [71,72]. The *SORL1* gene encodes for the SORL1 protein, a member of the vacuolar protein sorting (VPS10P) domain receptor gene family, with many emerging functions in neuronal-viability, signaling and intracellular trafficking [73]. Notoriously, this cargo protein is involved in the trafficking of amyloid beta precursor protein into recycling pathways, and, thus, it is protective against Aβ secretions: Specific rare variants in *SORL1* lead to the loss of this important function, altering the levels of Aβ peptides and interfering with APP trafficking [74]. Of note, alterations in cholesterol efflux and endocytic pathways have been already described in FTD [75,76]. According to bioinformatics analysis, Patient number 7 showed a potential damaging *SORL1* mutation (c.C2185T, p.R729W). In particular, the mutation is localized within the VPS10P domain and results in a potentially disruptive substitution due to the largely different sterical and chemical properties of tryptophan. The in silico mutagenesis of the arginine in position 729 in tryptophan was identified (with 41.4% confidence) to be the most likely tryptophan rotamer included in the mutated protein. The mutation causes a visible effect on nearby binding forces and, consequently, the 3D structure. Other variants falling in the VPS10P domain have been reported in literature to be potentially pathogenic, strengthening our finding [77].

Finally, mutations in *LRRK2* and *ALS2* genes have been reported in early-onset, sporadic form of PD and in patients with MND, respectively [78,79,80].

The *LRRK2* gene encodes the dardarin protein, active in the brain, which presents specific leucine-rich regions important for protein–protein interactions. Overall, it regulates various processes, including autophagy, immune response, neurite outgrowth and vesicle trafficking [81]. Probably, mutations contribute to alter one or more specific pathways, including vesicle trafficking [82,83]. Interestingly, a correlation between specific *LRRK2* variants and the circulating levels of the progranulin protein was highlighted by Caesar et al. [84]. We revealed a rare variant (c.T4937C, p.M1646T) in Patient 7 that is predicted to be tolerated by in silico prediction, even if the gene shows a negative RVIS.

The *ALS2* gene encodes for alsin, a protein playing crucial roles in the maintenance and survival of neurons. Precisely, alsin acts as a guanine nucleotide exchange factor for the small GTPase Rab5, modulating endocytic, biosynthetic and autophagic pathways [85]. It is noteworthy that these processes are also altered in FTD [82,86], thus suggesting that a role of *ALS2* in FTD aetiology is plausible. We identified an extremely rare variant (rs200950390, p.A517G) in Patient 7. The CADD tool suggested that this mutation might be pathogenic. Of note, this variant is fivegenes, i.e., *SORL1*, *FUS*, *LRRK2*, *ALS2* and *ABCA7*. These data are in line with our findings in early onset dementia patients and with the recently reported evidence, suggesting that sporadic forms of FTD could represent a polygenic disorder where multiple pleiotropic loci contribute to disease risk [32,87]. Interestingly, patient symptoms progressed very rapidly over a brief period, with a global clinical dementia rating scale changing from 1 (mild dementia) to 3 (severe dementia) in twelve months. This severe clinical phenotype could be explained, at least partially, by the presence of multiple mutations in the genome: It was reported that a rare variant can have severe effects on clinical phenotype in terms of broader somatic impact, greater severity, and earlier onset, compared to other types of more frequent mutations [59,88]. In this subject, five rare variants have been identified in five different genes. In this regard, we can speculate that different mutations in multiple genes may have influenced one or more disease pathways with a synergic outcome, worsening the progression of the clinical symptoms.

In this study, we have provided new insights into the molecular mechanisms underlying early onset FTD. We revealed that the 50% of sFTD patients showed at least one rare missense variant in AD, PD, ALS and LBD-associated genes, confirming the genetic pleiotropy in these neurodegenerative diseases. In parallel, a synergic genetic effect on FTD of the investigated genes has been suggested by the presence in one single patient of variants in five different genes. This finding is in line with recently reported evidence in early onset dementia patients [87,89]. Though most of these genes are not notoriously involved in FTD, they play a key role in multiple cellular pathways, including neuronal-viability and survival, inflammatory response, energy metabolism, phospholipid and cholesterol efflux, intracellular and vesicle trafficking, which are notoriously compromised in FTD. It is noteworthy that 50% of FTD cases did not carry any variants in the all screened genes, revealing a still missing heritability. This result is not surprising given the extreme aetiopathological complexity of these neurodegenerative disorders, where further potential molecular actors could be involved. 

Further studies in larger cohorts, employing a genome wide approach as well as a targeted approach with a focus on the herein identified pathways might highlight pathogenic variants in novel genes explaining the missing heritability of FTD.

## 4. Materials and Methods

A schematic diagram representing the whole work and steps/tool employed in this study is reported in Figure S2.

### 4.1. Subjects

From a larger FTD cohort [10], we selected a homogeneous group of eight patients (five females and three males) with a clinical diagnosis of FTD, according to international guidelines [3,4,90]. Patients were selected on the base of three inclusion criteria: a) Subjects belonging to sporadic category, classified by using Wood’s classification criteria [9]; b) the absence of mutations in *C9orf72*, *MAPT* and *GRN*; and c) a reported age of onset below 65 years. Clinical and demographic characteristics are shown in Table 1.

Blood samples were collected from all patients, and genomic DNA (gDNA) was obtained according to standard procedures. Patients provided written informed consent. The study was approved by the local ethical committee (Prot. N. 44/2016, 8/2017, 111/2017).

### 4.2. Next Generation Sequencing and Sanger Sequencing Analyses

gDNA samples from sFTD patients were analyzed on Illumina Miseq instrument using a Trusight One panel, characterized by a global gene list of 4.813 clinically relevant genes, harboring disease-causing variants including dementia-associated genes (Illumina, Inc., San Diego, CA, USA). According to the protocol, 5 ug of each gDNA were used to prepare sequencing libraries using TruSight One Sequencing Panel Library Prep Kit (Illumina, Inc., San Diego, CA, USA), according to manufacturer’s instructions. The size, quantity and quality of the libraries were assessed by the High Sensitivity DNA Chip on the Bioanalyzer instrument (Agilent Technologies, Santa Clara, CA, USA). The obtained sequence reads were aligned to the hg19 human reference sequence using the Burrow–Wheeler Aligner (BWA version 0.7.12). Duplicated reads were removed with Picard tools (http://broadinstitute.github.io/picard/). Local realignment, recalibration, and variant calling were conducted with the Genome Analysis Tool Kit (GATK version 3.30) [91]. To confirm the effective presence of the identified variants, Sanger sequencing was performed by using the automated ABI3130xl DNA Analyzer (Applied Biosystems, Foster City, CA, USA).

### 4.3. Prioritization and Validation of NGS Data

To facilitate variants filtering, two approaches were considered: a) Variants within FTD- and related disorders-associated genes (e.g., AD; PD; ALS; MND; LBD), as reported in literature data; b) coding variants (e.g., insertions/deletions frameshift, stop gain/stop loss and missense variants) characterized by a minor allele frequency (MAF) ≤ 0.01 in the exome aggregation consortium (ExAC, http://exac.broadinstitute.org/) database. All the identified NGS variants were annotated according to: a) Type of mutations (synonymous; non-synonymous; ins/del non-frameshift; ins/del frameshift; stop gain; stop loss); b) annotation in single nucleotide polymorphism database (dbSNP, rs number); and c) frequency in the ExAC database.

### 4.4. In Silico Prediction

The RVIS was downloaded (http://genic-intolerance.org) to evaluate the polymorphic variability of each mutated gene. Specifically, it represents a tolerance score in which more negative values express increasing intolerance of gene to mutations.

The impact of validated variants was predicted by using different bioinformatics programs. Specifically, these prediction tools were selected to evaluate the functional consequences at 3 different levels: Protein conformation and function (i.e., SIFT, Polyphen-2, Vest3, LR, MutationTaster), evolutionary conservation of both nucleotide variants (i.e., gerp, LRT, Phylop, SiPhy, FATHMM) and amino acid sequence (i.e., MutationAssessor). To define the deleteriousness effect of the identified variants, we considered two ensemble scores based on the simultaneous evaluation and integration of different independent bioinformatics scores: The CADD and the radial SVM.

In addition, for the variants that showed deleteriousness in both ensemble scores, we evaluated the effect of the variant on the protein function and 3D structure. Briefly, we used Elaspic [92] to evaluate if the variant fell on a functional domain, and then we used PyMol2 (https://pymol.org/2/) to estimate the effect of the variant on the residue binding forces in a radius of 5 Angstrom. The 3D crystal structure was downloaded from the RCSB protein data bank (http://www.rcsb.org/pages/publications).

## Figures and Tables

**Table 1:** Clinical and demographic characteristics of sporadic frontotemporal dementia (sFTD) patients included in the study.

Subject	Gender	Diagnosis	Age at Onset
1	M	PPA	51
2	M	bvFTD	48
3	F	FTD	50
4	M	PPA	55
5	F	FTD	53
6	F	PPA	57
7	F	FTD	63

M (male); F (female); PPA (primary progressive aphasia); bvFTD (behavioural frontotemporal dementia); FTD (frontotemporal dementia not otherwise sub-classified).

**Table 2:** Genes and rare variants identified in sFTD.

Subject	Chromosome	Reference Sequence	Gene	Exon	Nucleotide Variation	Aminoacidic Variation	Variant Frequency	dbSNP	Previously Associated Phenotypes
**1**	1	NM_001171811	*GBA*	7	G832A	E278K	0.01	rs2230288	Gaucher’s Disease; PD and parkinsonism; LBD
**5**	19	NM_019112	*ABCA7*	42	C5585A	P1862H	n.a.	n.a.	AD
**6**	1	NM_001123377	*PARK7*	6	A328G	T110A	1.00E^-04^	rs45577037	PD
**7**	19	NM_019112	*ABCA7*	39	A5389C	N1797H	n.a.	n.a.	AD
	16	NM_001170634	*FUS*	4	G235A	G79S	1.00E^-04^	rs776474571	ALS; FTD; ALS-FTD
	11	NM_003105	*SORL1*	16	C2185T	R729W	1.50E^-05^	n.a.	AD
	12	NM_198578	*LRRK2*	34	T4937C	M1646T	0.01	rs35303786	PD
	2	NM_020919	*ALS2*	6	C1550G	A517G	9.00E^-05^	rs200950390	ALS; MND

*GBA* (glucosylceramidase beta); *ABCA7* (ATP binding cassette subfamily A member 7); *PARK7* (Parkinsonism-associated deglycase 7); *FUS* (FUS RNA binding protein); *SORL1* (sortilin related receptor 1); *LRRK2* (leucine rich repeat kinase 2); *ALS2* (alsin Rho guanine nucleotide exchange factor); PD (Parkinson’s disease); LBD (Lewy body fementia); AD (Alzheimer’s disease); ALS (amyotrophic lateral sclerosis); FTD (frontotemporal dementia); MND (motor neuron disease); dbSNP (single nucleotide polymorphism database, rs number). Allele frequency of each variant was extracted from ExAC (exome aggregation consortium) database, and it is relative to non-Finnish European population.

**Table 3:** In silico prediction of the pathogenicity of each identified rare variant.

Subject	Gene Information		Variant Information		Deleterious Effect on Protein Conformation/Function					Evolutionary Conservation (DNA Sequence)					Evolutionary Conservation (AA Sequence)	Ensemble Scores	
	Gene Name	RVIS	Nucleotide Change	Aminoacidic Change	SIFT	Polyphen2 HDIV	VEST3	LR	Mutation Taster	Gerp++_RS	LRT	phyloP46way placental	phyloP100way vertebrate	FATHMM	Mutation Assessor	CADD phred	Radial SVM
1	*GBA*	0.18	G832A	E278K	T	B	P	D	A	P	N	D	N	D	L	N	D
5	*ABCA7*	−2.15	C5585A	P1862H	T	P	N	D	N	P	n.a.	N	N	D	L	N	D
6	*PARK7*	0.08	A328G	T110A	T	B	P	T	D	D	D	D	D	D	L	N	T
7	*ABCA7*	−2.15	A5389C	N1797H	D	P	N	D	N	P	n.a.	N	N	D	L	N	D
	*FUS*	−1	G235A	G79S	T	P	N	T	D	D	D	D	D	D	L	N	T
	*SORL1*	−2.34	C2185T	R729W	D	D	D	D	D	D	D	D	D	D	M	M	D
	*LRRK2*	−1.13	T4937C	M1646T	T	B	P	T	D	D	D	D	D	T	L	N	T
	*ALS2*	−1.46	C1550G	A517G	D	D	P	T	D	D	D	D	D	T	N	D	T

*Notes:* RVIS: Residual Variation Intolerance Score (represents a tolerance score in which more negative values express increasing intolerance of gene to mutations); SIFT: Scale-invariant feature transform (D: Deleterious; T: Tolerated); PolyPhen-2: Polymorphism Phenotyping v2 (D: Probably damaging; P: Possibly damaging; B: Benign); VEST3: Variant Effect Scoring Tool 3.0 (D: Probably damaging; P: Possibly damaging; N: Neutral); LR: Logistic regression (D: Deleterious; T: Tolerated); Mutation Taster (A: Disease causing automatic; D: Disease causing; N: Polymorphism); GERP++_RS: Genomic Evolutionary Rate Profiling Rejected Substitutions (D: Probably damaging; P: Possibly damaging); LRT: Likelihood ratio test (D: Probably damaging; N: Neutral; n.a.: not available); phyloP46way placental and phyloP100way vertebrate (D: Deleterious; N: Neutral); FATHMM: Functional Analysis Through Hidden Markov Models (D: Deleterious; T: Tolerated); Mutation Assessor (predicted functional: High (“H”) or medium (“M”); predicted non-functional: Low (“L”) or neutral (“N”)); CADD: Combined Annotation Dependent Depletion (N: Predicted not damaging; M: Moderately damaging; D: Strongly damaging, as reported by [Holstege et al. 2017]); radial support vector machine (SVM) based ensemble prediction score (D: Deleterious; T: Tolerated, as reported by [Dong et al. 2015]).

## Supplementary Materials

Supplementary materials can be found at https://www.mdpi.com/1422-0067/20/16/3903/s1.
