# Supplementary material for: The Missing Heritability of Sporadic Frontotemporal Dementia: New Insights from Rare Variants in Neurodegenerative Candidate Genes
Source: Int J Mol Sci. 2019 Aug 10;20(16):3903. doi: 10.3390/ijms20163903 (PMC6721049; doi:10.3390/ijms20163903)
Supplement: Supplementary file 1 [file ijms-20-03903-s001.zip › Ciani_Bonvicini_InJMolSciences_SupplementaryMaterial_Revised.docx]

# Table S1. List of screened genes.

| **GENE ACRONYM** | **GENE**  **FULL NAME** | **CHROMOSOME** |
| --- | --- | --- |
| *ABCA7* | ATP binding cassette subfamily A member 7 | 19 |
| *ALS2* | alsin Rho guanine nucleotide exchange factor | 2 |
| *ANG* | angiogenin | 14 |
| *APOE* | apolipoprotein E | 19 |
| *APP* | amyloid beta precursor protein | 21 |
| *ATP7A* | ATPase copper transporting alpha | X |
| *ATP7B* | ATPase copper transporting beta | 13 |
| *ATXN2* | ataxin 2 | 2 |
| *BDNF* | brain derived neurotrophic factor | 11 |
| ***C9orf72*** | chromosome 9 open reading frame 72 | 9 |
| ***CHMP2B*** | charged multivesicular body protein 2B | 3 |
| *CST3* | cystatin C | 20 |
| *CSTA* | cystatin A | 3 |
| *CSTB* | cystatin B | 21 |
| *DCTN1* | dynactin subunit 1 | 2 |
| ***FUS*** | Fused in sarcoma | 16 |
| *GBA* | glucosylceramidase beta | 1 |
| ***GRN*** | progranulin | 17 |
| *LRRK2* | leucine rich repeat kinase 2 | 12 |
| ***MAPT*** | microtubule associated protein tau | 17 |
| *MATR3* | matrin 3 | 5 |
| *NOTCH3* | notch receptor 3 | 19 |
| *OPTN* | optineurin | 10 |
| *PARK2* | parkin RBR E3 ubiquitin protein ligase | 6 |
| *PARK7* | Parkinsonism associated deglycase | 1 |
| *PINK1* | PTEN induced kinase 1 | 1 |
| *PRNP* | prion protein | 20 |
| *PSEN1* | presenilin1 | 14 |
| *PSEN2* | Presenilin2 | 1 |
| *SERPINA1* | serpin family A member 1 | 14 |
| *SETX* | senataxin | 9 |
| *SNCA* | synuclein alpha | 4 |
| *SOD1* | superoxide dismutase 1 | 21 |
| *SORCS1* | sortilin related VPS10 domain containing receptor 1 | 10 |
| *SORL1* | sortilin related receptor 1 | 11 |
| *SORT1* | sortilin 1 | 1 |
| *SPG11* | SPG11 vesicle trafficking associated, spatacsin | 15 |
| *SQSTM1* | sequestosome 1 | 5 |
| ***TARDBP*** | TAR DNA binding protein | 1 |
| *TBK1* | TANK binding kinase 1 | 12 |
| *TOMM40* | translocase of outer mitochondrial membrane 40 | 19 |
| *UBQLN2* | ubiquilin 2 | X |
| ***VCP*** | valosin containing protein | 9 |

*Note Table S1*: In bold, known FTD-related gene.


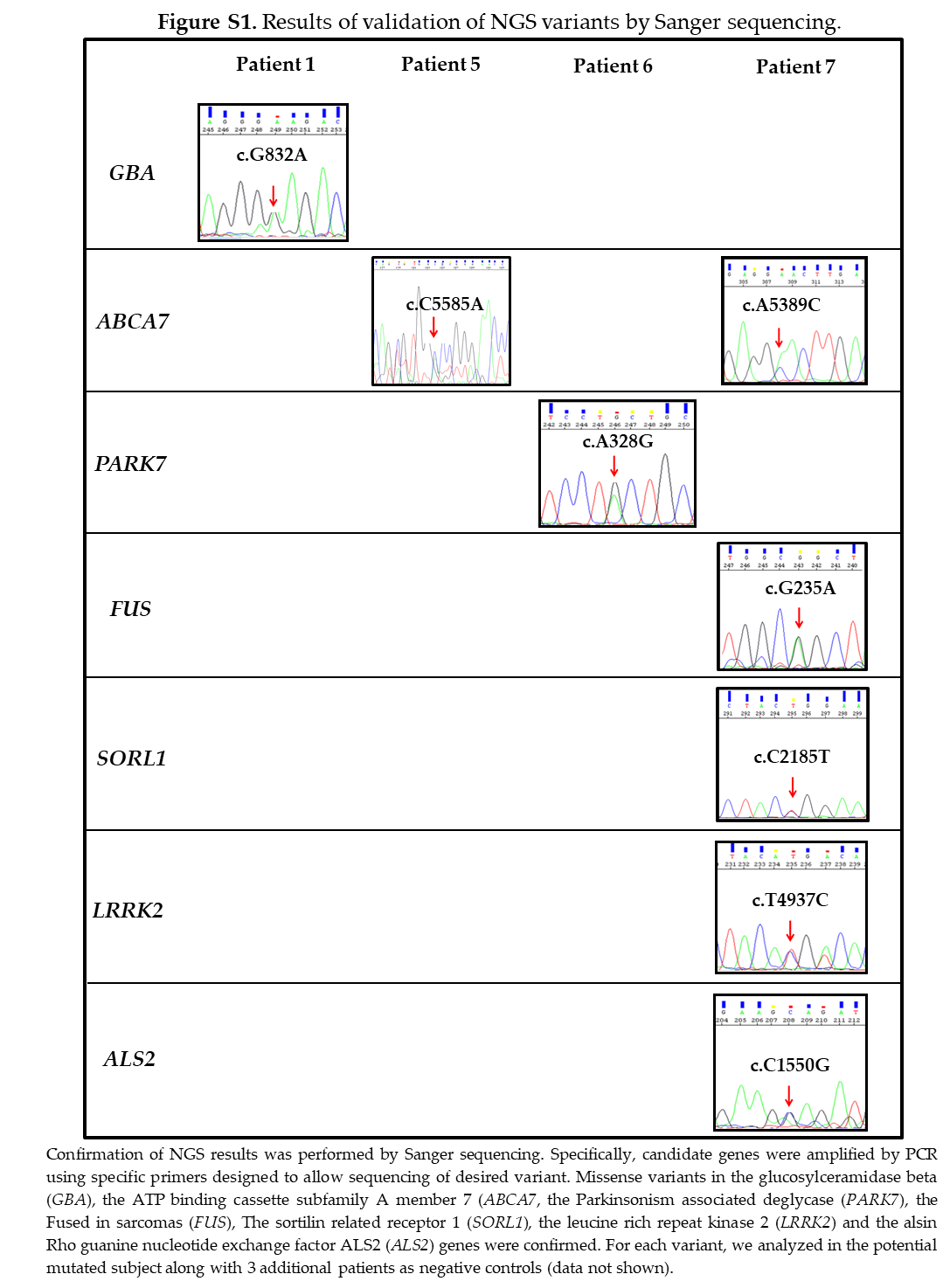
**Figure S1. Results of validation of NGS variants by Sanger sequencing.**


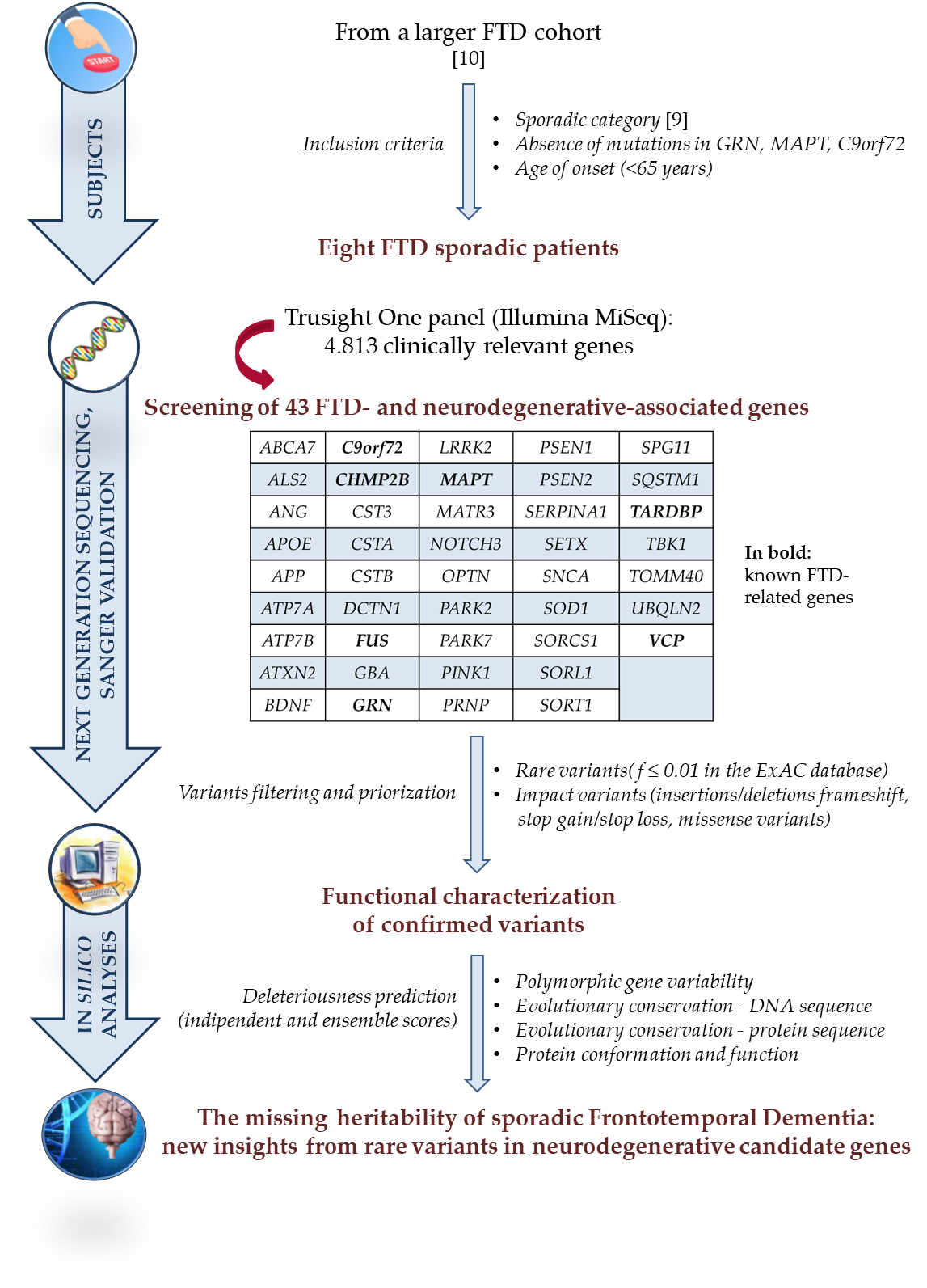
**Figure S2. The schematic diagram depicting the steps/tool used in the study.**
