# Supplementary figures and images for: The Missing Heritability of Sporadic Frontotemporal Dementia: New Insights from Rare Variants in Neurodegenerative Candidate Genes
Source: Int J Mol Sci. 2019 Aug 10;20(16):3903. doi: 10.3390/ijms20163903 (PMC6721049; doi:10.3390/ijms20163903)

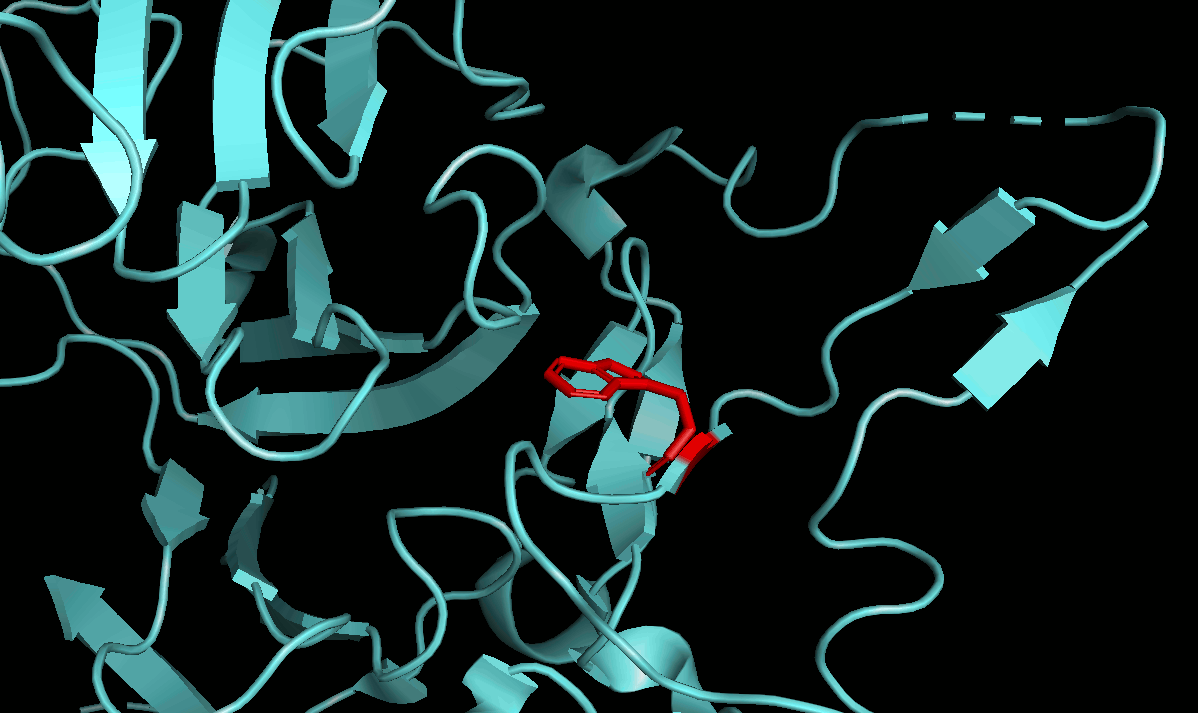

Supplement: Supplementary file 1 [file ijms-20-03903-s001.zip › Supplementary_video3_sorl1_vps10dom_wt_mut.gif]
